# Clonal Evolution of Myeloid Malignancies Treated With Microtransplantation: A Single‐Centre Experience

**DOI:** 10.1111/jcmm.70520

**Published:** 2025-03-24

**Authors:** R. Sammut, L. Fenwarth, A. Pelissier, A. Marceau, N. Duployez, S. Benachour, B. Dadone, T. Cluzeau, M. Loschi

**Affiliations:** ^1^ Département d'Hématologie Centre Hospitalier Universitaire de Nice Nice France; ^2^ Laboratoire d'Hématologie Centre Hospitalier Universitaire de Lille France; ^3^ Department of Biosystems Science and Engineering ETH Zurich Basel Switzerland; ^4^ Laboratoire Central d'anatomie et de Cytologie Pathologique Centre Hospitalier Universitaire de Nice Nice France; ^5^ INSERM U1065, Centre de Médecine Moléculaire Méditerranéen Université Nice Cote d'Azur Nice France; ^6^ Université Cote d'Azur Nice France

**Keywords:** acute myeloid leukaemia, bone marrow transplantation, chronic myelomonocytic leukaemia, clonal evolution, microtransplantation, myelodysplastic syndromes

## Abstract

Microtransplantation is a cellular therapy used in acute myeloid leukaemia and myelodysplastic syndromes as a maintenance therapy in patients ineligible for a regular allogeneic stem cell transplantation. We performed a monocentric retrospective study of acute myeloid leukaemia, myelodysplastic syndromes, and chronic myelomonocytic leukaemia patients who underwent microtransplantations at Nice University Hospital. We analysed the evolution of the disease mutational status after microtransplantation. We report 18 patients who underwent microtransplantation courses, with a total of 47 microtransplantations performed between February 2020 and June 2022. We observed long‐term remissions even in high‐risk patients. Founder mutations persisted throughout the follow‐up, whereas it was more variable for other nonfounder mutations, with most of the nonfounder mutations variant allele frequency decreasing over time. Safety data were reassuring; no graft versus host disease was recorded, and cytokine release syndromes were manageable. Relapses or progressions were associated with the emergence or increase of a *TP53* mutated clone. Microtransplantation is a promising therapy for patients ineligible for regular allogeneic stem transplantation. Further larger and randomised studies are required to establish its place as a maintenance therapy in myeloid malignancies.

## 1 Introduction

Allogeneic stem cell transplantation (ASCT) is the first‐choice consolidation therapy for patients with intermediate and high risk acute myeloid leukaemia (AML) and is also the only curative treatment for patients with myelodysplastic syndromes (MDS) and chronic myelomonocytic leukaemia (CMML) [1]. Despite the development of reduced intensity conditioning (RIC) and nonmyeloablative conditioning (NMA), performing ASCT for elderly patients with or without comorbidities is linked to an increased nonrelapse mortality and therefore excludes such patients from the opportunity to receive a cellular therapy. These subgroups of patients, who have an indication for transplantation but are too frail to undergo the procedure, have a very poor outcome with a high risk of relapse [2]. After obtaining complete remission (CR) in AML patients, performing a maintenance therapy with hypomethylating agents (HMA) [3, 4, 5] or a targeted therapy [6] could be part of the solution to prevent relapse. Oral Azacitidine (AZA) as well as targeted therapies for *FLT3* [6] and *IDH1/IDH2* [7] mutated patients are for now the only approved treatments in this indication after intensive chemotherapy [3]. For patients with chronic myeloid malignancies such as MDS and CMML, HMA are often used until progression, and despite the use of new therapies such as venetoclax [8] and targeted therapies [9], the outcomes of patients with contraindications to ASCT remain dismal.

Microtransplantation (MT) is a non‐engrafting alloreactive stem cell therapy based on the infusion of an HLA mismatched stem cell graft from a haploidentical donor, immediately after cytotoxic therapy and without immunosuppressive treatment. It is a consolidation therapy usually performed repeatedly and associated with cytarabine‐based regimens for AML and HMA‐based regimens for MDS patients [10, 11]. Although the immunological mechanisms underlying the efficacy of microtransplantation are not completely elucidated, it has been reported that the infusion of HLA mismatched cells induces both a graft and a host antileukaemia effect responsible for a systemic cytokine reaction [12]. Because there is no engraftment, there is no sustained chimerism and graft versus host disease (GvHD) is rare. In elderly patients with de novo AML, a trial randomising MT versus chemotherapy alone in consolidation has been published by Guo et al. in 2011 [13]. MT was administrated during induction and in postremission therapy. The MT group had a higher rate of CR (80.0% vs. 42.8%; *p* = 0.006) and a better 2‐year overall survival (OS) (39.3 vs. 10.3%; *p* = 0.006). A phase II international multicentre study demonstrated a better efficacy among patients who received at least 2 MT and who had a favourable or intermediate disease [14]. Despite these encouraging results, no other randomised trials studying MT have been performed since, and the place of MT among elderly patients with high risk of relapse remains unclear. Very few data are available on the clonal evolution in patients receiving MT as a consolidation treatment. Here, we describe our experience at the Nice University Hospital using MT as consolidation therapy for patients with high and intermediate risk AML, MDS and MDS/MPN who are ineligible for ASCT.

## 2 Materiel and Methods

### 2.1 Patients and Donors

We retrospectively collected data from patients with AML, high‐risk MDS or CMML who started MT consolidation courses from February 2020 to June 2022. The cut‐off date for follow‐up was 15 September 2022. This retrospective study included data collected from the Promise European Bone Marrow Transplantation (EBMT) database registry (CIC523). Patients selected for MT had an indication of ASCT but were deemed ineligible for a regular allogeneic transplantation due to age and/or comorbidity. The risk of relapse and indication of ASCT was determined according to the European Leukaemia Net (ELN) 2017 classification [15] for AML patients, and according to the revised (International Prognostic Scoring System) IPSS [16] for MDS patients. For CMML patients, the indication of ASCT was based on bone marrow excess blasts > 10%, corresponding to CMML‐2 according to the World Health Organisation (WHO) 2016 definition [17] and risk of relapse was assessed by the CMML‐Mol score [18]. ASCT contraindication was established by a multidisciplinary committee, based on patients' age, performance status, comorbidities and the Haematopoietic Cell Transplantation‐specific Comorbidity Index (HCT‐CI) score [19]. Response was defined according to the 2018 International Working Group (IWG) MDS response criteria [20], 2016 IWG CMML response criteria [21] and 2003 IWG AML response criteria [22]. In order to homogenise disease status among patients, we combined marrow response and complete remission with incomplete count recovery (CRi).

Donors were adult haploidentical related donors, children, or siblings of the patient. They underwent leukapheresis twice, on a 2‐day span. Leukapheresis was performed after 5 days of stimulation by Filgrastim 5 μg/kg BID.

All patients gave signed informed consent for data collection and subsequent analysis. This study was conducted in accordance with the principles of the Declaration of Helsinki.

### 2.2 Treatment

#### 2.2.1 Previous Treatment

Intensive chemotherapy consisted of Cytarabine and Anthracycline induction therapy usually followed by Intermediate dose Cytarabine based consolidation or CPX‐351 induction and consolidation. Patients not fit enough to pursue the intensive chemotherapy regimen after induction shifted to an HMA based regimen. The HMA treatment was Azacitidine (AZA) at the dose of 75 mg/m2 a day during 7 days or 5 + 2 days and was for some patients associated with either Venetoclax (400 mg/day) or Pevonedistat (IV injection at 20 mg/m^2^ at D1, D3, D5), or with Eprenetapopt (IV injection of 4500 mg for 4 days) for patients with *TP53* mutation. If they were previously treated by AZA based therapy, patients began their MT courses after receiving at least 3 courses of pre‐MT treatment. For MDS and CMML, patients could receive a first round of MT course being treatment naïve if they had a stable disease. Patients with AML should be in complete remission (CR) or in CRi; patients with MDS or CMML should have at least a stable disease without haematological improvement (HI).

#### 2.2.2 Microtransplantation Course

**FIGURE 1:**
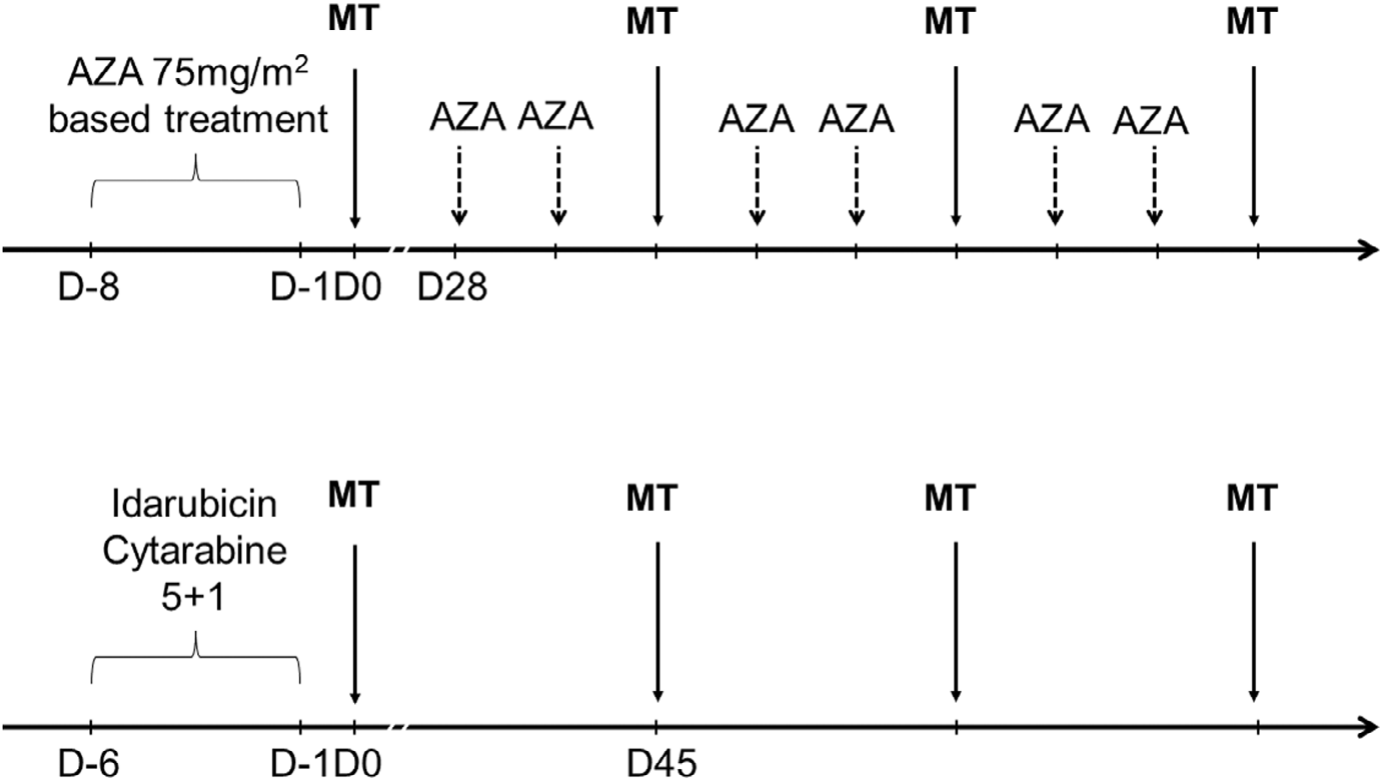
Microtransplantation treatment plan. Cytotoxic treatment associated with MT was azacytidine‐based treatment for patients previously treated with HMA or treatment naïve, or Idarubicin and Cytarabine (5 + 1) for patients previously treated with intensive chemotherapy. AZA azacytidine; MT microtransplantation; HMA hypomethylating agents.

The administration of HLA mismatched peripheral blood (PB) stem cells was always associated with a cytotoxic treatment. For patients who were previously treated with an HMA‐based regimen, the treatment was continued as such but followed by a MT. Patients then continued their treatment with HMA, one cycle every 28 days. MT course was performed every 3 cycles. For patients who were previously treated by intensive chemotherapy, the cytotoxic regimen used before MT was a subcutaneous 120 mg/m^2^ cytarabine injection for 5 days and IV 9 mg/m^2^ Idarubicin injection for 1 day (5 + 1 treatment). For such patients, four mini‐consolidations of Cytarabine and Idarubicin were planned. The HLA mismatched PB cells infusion was performed a day after the end of the cytotoxic treatment. The count of CD34+ cells had to be inferior to 5 × 10 [6]/kg. Patients were hospitalised before the start of the chemotherapy and discharged the day after their blood counts recovery. Patients received antifungal prophylaxis consisting of posaconazole for patients not receiving venetoclax and liposomal amphotericin B for patients receiving venetoclax. Three or 4 MT were initially planned for each patient, depending on the donor's PB haematopoietic stem cells (HSC) harvest. Patients did not finish their MT course if they relapsed from CR or had a progression of their disease or if they became unfit to pursue the treatment. The treatment plan is illustrated in Figure 1. We reported adverse events occurring during the MT course according to Common Terminology Criteria for Adverse Events (CTCAE) v5.0 [23].

### 2.3 NGS Analysis

Bone marrow exploration with molecular analysis was performed before any new MT. Capture‐based next‐generation sequencing (NGS) analysis of myeloid malignancies using a panel of 90 genes was performed in the Lille University Hospital. Mutations were considered positive if their respective variant allele frequency (VAF) was superior to 2%. *NPM1* measurable residual disease (MRD) was measured with TaqMan PCR for classical forms and digital PCR for rare forms.

### 2.4 Statistical Analysis

Statistical analyses were performed using the R program. Survival analyses were made using Kaplan–Meier estimation. We used the Bradley–Terry pairwise model to determine the dominant order of mutations and the evolution of each mutation during the treatment course. A mutation was considered to have occurred earlier and therefore dominant regarding another mutation if its VAF was ≥ 5%. VAF differences < 5% were too ambiguous to be considered positive in the model. Only patients with 2 mutations or more were included in the clonal evolution analysis. In the increase and decrease model, VAF variations ≥ 10% were considered significant in the analysis. VAF cut‐offs for apparition, increase, and decrease of mutations were determined according to Benard et al.'s clonal evolution analysis [24]. Fishplots were created using the chrisamiller/fishplot package [25]. Plots were produced using R programs and Graph Prism 10.0.3.

## 3 Results

### 3.1 Characteristics

**FIGURE 2:**
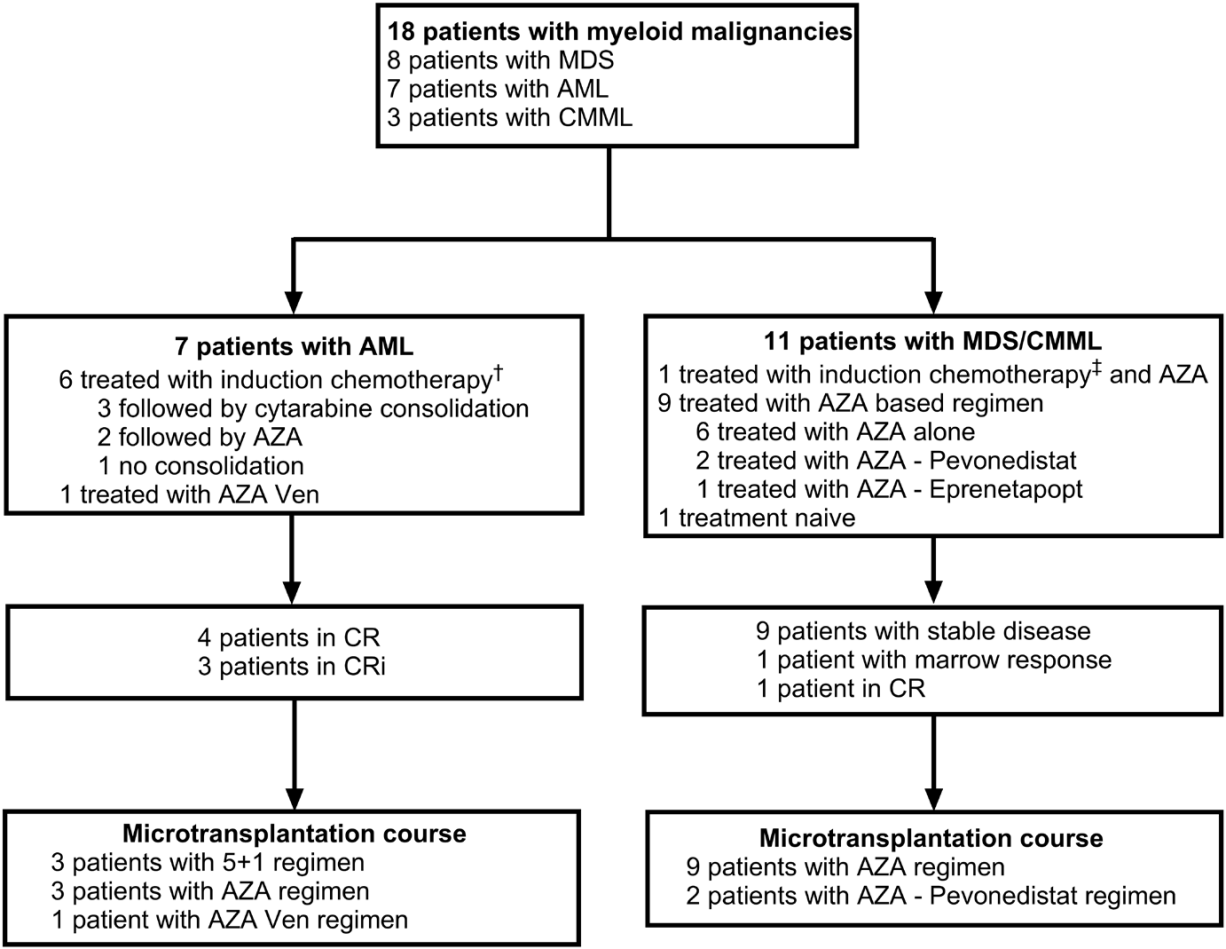
Flow chart. ^†^Induction therapy in AML patients consisted of an anthracycline and cytarabine combination and CPX‐351. ^‡^Induction therapy in MDS/CMML patients consisted of CPX‐351. AML acute myeloid leukaemia; MDS myelodysplastic syndrome; CMML chronic myelomonocytic leukaemia; AZA azacytidine.

Between February 2020 and June 2022, eighteen patients underwent MT courses at our institution and a total of 47 MT were performed. Patients were 43 to 88 years old, with a median age of 72.3 years old [IQR: 70.6–74.7] at the time of the first MT. The most common malignancy was MDS (8 patients), followed by AML (7 patients) and CMML (3 patients). All MDS patients were MDS with type 2 excess of blasts (MDS‐EB2), and all CMML patients were CMML‐2. The median HCT‐CI score was 2.5 [IQR: 0.25–3.75]. At the time of the first MT, four/7 patients with AML were in CR, and three/7 patients with AML were in CRi. Among patients with MDS and CMML, nine/11 patients had a stable disease, one/11 patient had a marrow response and one/11 patient was in CR. Previous treatment included anthracycline and cytarabine based induction for 5 patients, CPX 351 for 2 patients, AZA based treatment for 13 patients, AZA alone for 9 patients, AZA plus Pevonedistat for 2 patients, AZA plus Venetoclax for 1 patient and AZA plus Eprenetapopt for 1 patient. Three patients were treated with AZA after having received induction by intensive chemotherapy. One patient with CMML was treatment naïve and received MT as a frontline treatment. Previous treatment and status at baseline are represented in Figure 2. Donors were mismatched related donors with a compatibility of HLA 5/10 for 14 patients, 6/10 for 3 patients and 7/10 for 1 patient. Data are detailed in Table 1.

**TABLE 1:** Patients characteristics at the time of the first microtransplantation.

Characteristics		Patients (%)
Age	Median [IQR]	72.3 [70.6–74.7]
Sex	Male	12 (66.7)
Haemopathy	AML	7 (38.9)
	MDS	8 (44.4)
	CMML	3 (16.7)
ELN 2017	Adverse	3 (42.9)
	Intermediate	4 (57.1)
IPSSr	Very high	1 (12.5)
	High	7 (87.5)
CMML‐Mol	High	1 (33.3)
	Intermediate‐2	2 (66.7)
HCT‐CI	Median [IQR]	2.5 [0.25–3.75]
Previous treatment	Intensive chemotherapy^a^	4 (22.2)
	Intensive chemotherapy—AZA^b^	3 (16.7)
	AZA based	10 (55.6)
	AZA alone	6 (33.3)
	AZA—pevonedistat	2 (11.1)
	AZA—venetoclax	1 (5.6)
	AZA—eprenetapopt	1 (5.6)
	No previous treatment	1 (5.6)
Prior number of AZA cycles	Median [IQR]	4 [3.0–8.0]
Time from diagnosis to transplant (months)	Median [IQR]	6.4 [5.0–10.9]
Status before transplant	CR	5 (27.8)
	CRi/Marrow response	4 (22.2)
	Stable	9 (50.0)

Abbreviations: AML acute myeloid leukaemia, AZA azacytidine, CMML chronic myelomonocytic leukaemia, CR complete remission, CRi complete remission with haematological improvement, ELN European LeukemiaNet, HCT‐CI Haematopoietic Cell Transplantation‐specific Comorbidity Index, IPSSr Revised International Prognostic Scoring System, IQR interquartile, MDS myelodysplastic syndromes, MPN myeloproliferative neoplasms.

^a^ Intensive chemotherapy consisted of 7 + 3 induction with or without an intermediate dose of cytarabine, CPX‐351 induction with or without consolidation.

^b^ Intensive chemotherapy induction followed by azacitidine regimen.

### 3.2 Molecular and Cytogenetic Characteristics

Table 2 resumes the molecular characteristics of the patients at baseline. Among patients with AML, three had an adverse risk and four had an intermediate risk according to ELN 2017 risk stratification. Among patients with MDS, one patient was classified as very high risk, and seven patients were classified as high risk. In CMML patients, one patient was classified as high risk, and two patients were classified as intermediate‐2. One patient had a complex karyotype, two patients had a monosomal karyotype and 12 had a normal karyotype. Epigenetic modifier mutations were often present at the time of the first transplantation, such as *ASXL1* (6 patients), *TET2* (5 patients) and *DNMT3A* (4 patients). *RUNX1* was also one of the most frequent mutations at baseline (4 patients). *TP53* mutations were present in 2 patients at baseline. One patient had a *DDX41* germline mutation.

**TABLE 2:** Mutational status at baseline.

Functional class	Mutation	Patient
Epigenetic modifiers	*ASXL1*	6
	*DNMT3A*	4
	*TET2*	5
	*EZH2*	3
	*IDH2*	3
	*BCOR*	2
	*BCORL1*	1
Spliceosome complex	*SRSF2*	3
	*ZRSR2*	2
	*U2AF1*	2
Cohesin complex	*STAG2*	3
	*RAD21*	1
	*SMC1A*	1
	*SMC3*	1
Signalling, kinases pathways	*KRAS*	2
	*NRAS*	1
Nucleophosmin	*NPM1*	2
Transcription factors	*RUNX1*	4
Tumour suppressors	*TP53*	2
	*PHF6*	1

### 3.3 Response and Survival

The median follow‐up was 15.9 months [IQR: 9.95–23.68]. The median time between diagnosis and the first MT was 6.4 months [IQR: 5.0–10.9] and the median time between each MT was 2.8 months [IQR: 2.6–3.0]. The median number of MT performed per patient was 3 [IQR: 2–3]. Fifteen patients received an AZA‐based regimen with MT, three patients received a 5 + 1 regimen. The median count of CD34 and CD3 infused per MT was respectively 2.08 × 10 [6]/kg [IQR: 2–2.42] and 13.47 × 10 [7]/kg [IQR: 10.15–17.48].

**FIGURE 3:**
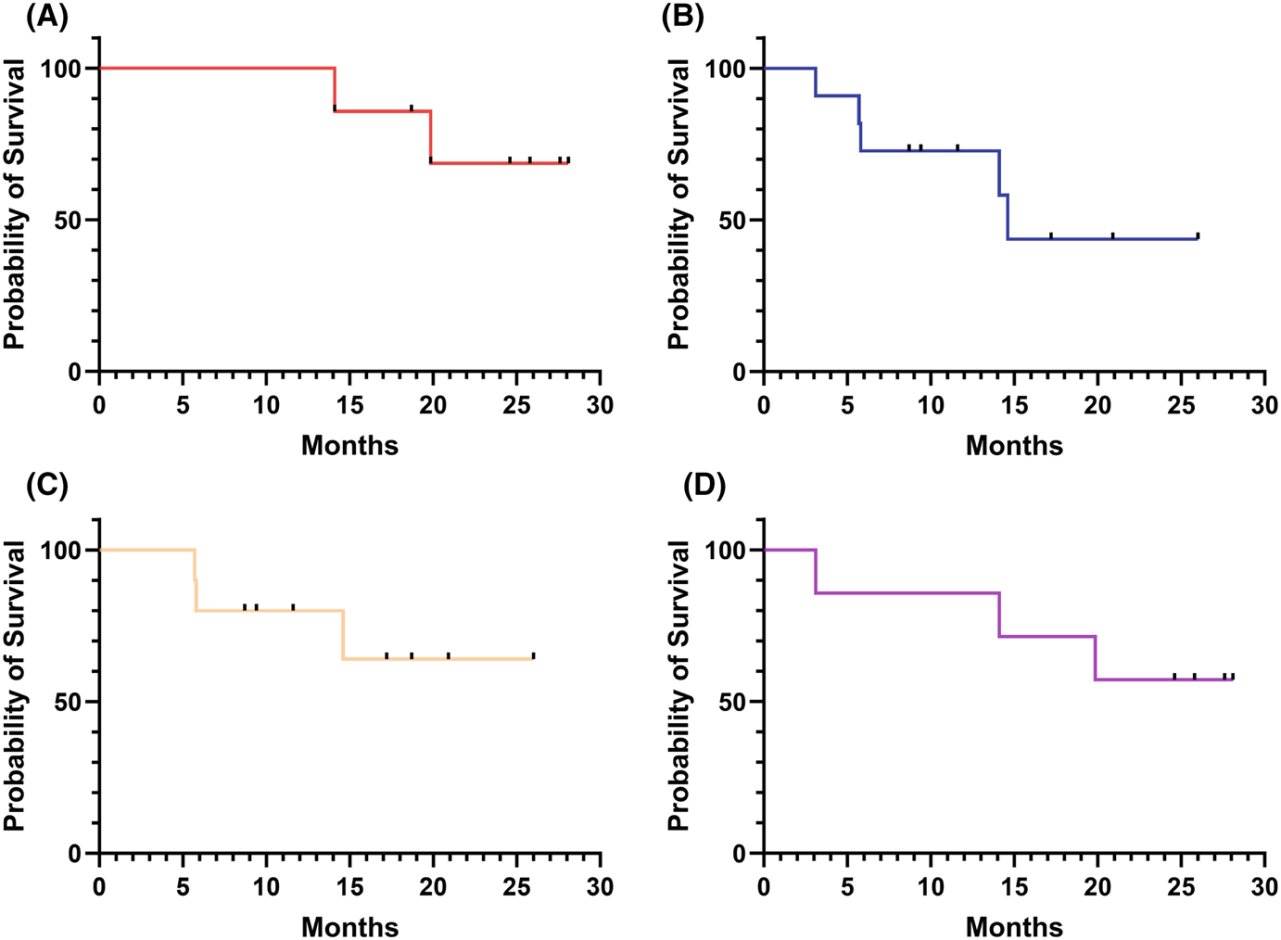
Survival probability. (A) Survival probability of AML patients. Median survival was not reached in this group. (B)Survival probability of MDS/CMML patients. Median survival estimated at 14.6 months. (C) Survival probability of patients previously treated by intensive chemotherapy. Median survival was not reached in this group. (D) Survival probability of patients previously treated by AZA‐based regimen. Median survival was not reached in this group. AML Acute Myeloid Leukaemia; MDS Myelodysplastic Syndromes; CMML Chronic Myelomonocytic Leukaemia; AZA Azacytidine.

We observed an improvement of response in 12 patients (77%) at the end of the MT course. Median OS was not reached in AML patients and was measured at 14,6 months [95% CI: 5.7–14.6] in MDS/CMML patients (Figure 3A,B respectively). According to prior treatment, median OS did not differ for patients who received either intensive chemotherapy or AZA‐based chemotherapy before MT (Figure 3C,D).

All seven patients with AML were in CR/CRi at the beginning of the treatment, and all patients stayed in CR during follow‐up except for one. This patient had a *DDX41* germline mutation, reached a first CR after a 7 + 3 regimen, but because of severe complications following the induction course, he received azacitidine as consolidation therapy associated with 4 MT. Unfortunately, he relapsed 38 months after AML diagnosis. We observed a molecular relapse among one of the youngest patients of the cohort despite 2 MT. This patient underwent an ASCT with a matched sibling donor (MSD) and died from a stage IV gut acute GvHD 2 months after the transplantation. One patient with AML stopped after 2 MT while being in CRi, continued AZA plus venetoclax treatment and was still in CRi at the end of the follow‐up.

Among AML patients who stopped after 3 MT, both of them remained in CR at the end of the follow‐up. One of them stopped the MT program because of his alcoholic cirrhosis and had two additional AZA cycles; the other patient had no other maintenance therapy and stayed in CR. One patient with an adverse risk AML completed 4 MT courses and is still in CR 33 months after the diagnosis of AML.

Among MDS/CMML patients, two patients were in CR at the end of the follow‐up, two were in marrow CR, three had stable disease, three had a progression of their disease and one relapsed from CR. Two patients stopped after receiving 1 MT: one patient with MDS had a consistent stable disease but had a decline in his performance status after the first MT, and supportive care was initiated; one patient with CMML did not continue the MT due to progression of the initial disease and died 3 months after the last MT. Three patients received 2 MT: one patient with MDS with stable disease at the beginning of the treatment obtained CR after the first MT, then relapsed from CR after the second MT and died of relapse 3 months after the last MT. One patient with CMML had a stable disease at the end of the treatment, continued AZA alone and experienced a progression into AML 5 months after the last MT. One patient with MDS was in marrow CR at the end of the treatment and continued AZA alone as well and stayed in marrow CR at the end of the follow‐up. Five patients stopped after 3 MT: two of them had consistent stable disease and were respectively treated with an IDH2 and an IDH1 inhibitor, one obtained marrow CR and continued treatment with AZA—Pevonedistat and two patients obtained CR and did not continue any antileukaemic treatment at the end of the follow‐up. One patient with MDS completed the 4 MT planned and stayed in stable disease all along but had a progression into AML 2 months after ending the last MT.

### 3.4 Clonal Evolution, Association With Response

**FIGURE 4:**
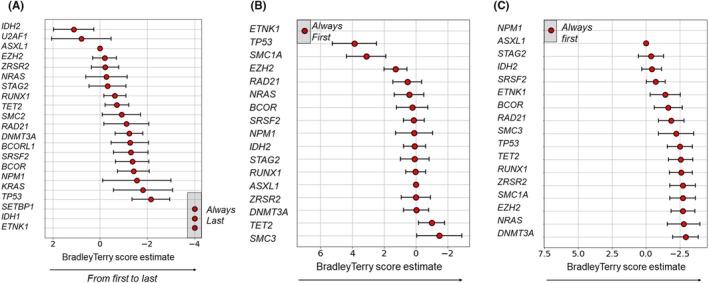
Bradley Terry model. (A) Apparition of mutation. From first to appear to last, at baseline. Points on the left tend to start mutating before other genes. Grey square represents nonsignificant result. (B) Increase of mutation. VAF 10% up after first time point. From first to last, during MT. Grey square represents nonsignificant result. (C) Decrease of mutation. VAF 10% down after first time point. From first to decrease to last, during MT. Grey square represents nonsignificant result. MT microtransplantation; VAF variant allele frequency.

We obtained NGS follow‐up for 12 patients. Variations of VAFs are reported in Figure S1. Six patients were in cytologic CR at the last NGS follow‐up. Among patients in CR, we observed one *NPM1* MRD clearance with stability of the other mutations. Another patient had a molecular relapse detected by an increased *NPM1* MRD. Two out of five relapses or progression had an NGS follow‐up during their MT course. Each of them had an increase of the *TP53* VAF. *TP53* was present at diagnosis or appeared at progression in 3 out of 5 cases of relapse or progression. We observed 4 patients with stable disease at the last NGS follow‐up. Eleven patients were analysed for the apparition of the Bradley Terry model. In our patients, *IDH2, U2AF1* and *ASXL1* were the first mutations to occur. These mutations are involved in spliceosome complex and DNA methylation. *ETNK1*, *IDH1* and *SETBP1* were the last mutations to appear but not significantly. These mutations were present in only one patient in the analysis, and *IDH1* and *ETNK1* appeared during follow‐up. *TP53, KRAS and NPM1* were the latest to appear significantly. During treatment, *NPM1* was the first mutation to decrease but not significantly, followed by *ASXL1* and *STAG2*. *ETNK1* was the first mutation to increase during treatment but not significantly, followed by *TP53* and *SMC1A*. The order of occurrence of mutations, and the order of increase and decrease of mutations during treatment are detailed in Figure 4.

We represented clonal evolution of two patients using a fishplot, in Figure S2: patient 2 in constant CR, with stable *IDH2* and *SRSF2* mutations, and a disappearance of the *NPM1* mutation between the first MT and after treatment. *IDH2* was considered a clonal mutation, and *SRSF2* and *NPM1* were considered subclonal mutations, according to the Bradley model. Patient 15 had a progression of CMML into AML after one MT. According to our findings, *TP53* was a subclonal mutation, and *ZRSR2*, *TET2, EZH2, SMC1A*, *ASXL1* and *RUNX1* were considered clonal mutations. The patient had a very low burden of the *DNMT3A* clone (with a VAF at 2% at first MT) which we did not represent on the fishplot.

### 3.5 Safety

Seven patients died during the follow‐up; no death was attributed to the MT procedure: five patients died from relapse or progression, one patient died from alcoholic cirrhosis 13.5 months after the last MT while being in CRi and one patient died of GvHD after undergoing ASCT. The most common adverse events were anaemia, neutropenia, thrombocytopenia, and fever (Table 3). Thirty‐one microtransplantations were followed by a cytokine release syndrome (CRS); we observed only one grade 3 CRS and no grade 4. No case of GvHD proven by biopsy was confirmed in our patients. Seven infusions were followed by a cutaneous rash associated with fever, appearing within 24 h after the infusion, and having a spontaneous resolution, suggesting that it was also related to CRS and not related to a GvHD. We reported 4 cases of documented infection, including 3 catheter‐related infections. Two patients had undocumented pneumonitis during MT. We observed an asymptomatic Epstein Barr Virus (EBV) reactivation in one patient all along his 4 MT that did not require treatment. A Cytomegalovirus (CMV) primo infection transmitted by the graft was successfully treated with ganciclovir in one patient. Safety data are detailed in Table 3.

**TABLE 3:** Adverse events during microtransplantation courses.

AE	All grade *n* (%)	Grades 3–4 *n* (%)
Anaemia	45 (93.8)	16 (33.3)
Neutropenia	42 (87.5)	36 (75.0)
Thrombocytopenia	43 (89.6)	31 (64.6)
Hematoma	2 (4.2)	—
CRS	31 (64.6)	1 (2.1)
Rash	7 (14.6)	—
Febrile neutropenia	27 (56.3)	27 (56.3)
Sepsis	3 (6.3)	3 (6.3)
Catheter related infection	4 (8.3)	4 (8.3)
Pneumonitis	2 (4.2)	2 (4.2)
EBV reactivation	1 (2.1))	—
CMV reactivation	1 (2.1)	—
HSV reactivation	1 (2.1)	—
Constipation	12 (25.0)	3 (6.3)
Diarrhoea	5 (10.4)	—
Nausea	4 (8.3)	—
Transaminitis	2 (4.2)	—
AKI	3 (6.3)	3 (6.3)
Fall	2 (4.2)	—
Syncope	1 (2.1)	1 (2.1)
Thromboembolic event	1 (2.1)	—

*Note:* Adverse events were reported according to Common Terminology Criteria for Adverse Events (CTCAE) Version 5.0.

Abbreviations: AE adverse events; CRS cytokine release syndrome; EBV Epstein Barr Virus; CMV cytomegalovirus; HSV herpes simplex virus; AKI acute kidney injury.

## 4 Discussion

We observed sustained remission rates among elderly patients with AML even among patients with high‐risk ELN. We observed only one relapse in AML patients. In MDS/CMML patients, results are more diverse: we observed two CRs, two marrow CRs, three stable diseases, three progressions and one relapse from CR at the end of the follow‐up. Most patients with MDS/CMML had stable disease at the beginning of MT treatment.

It may suggest that the immunotherapy effect of MT may not be sufficient to produce long term CR among patients with MDS/CMML but may contribute and consolidate an already established remission. Survival analyses were also encouraging, with a median OS not reached in the overall population, as well as in the AML group, and in both the AZA‐based treatment and the intensive chemotherapy group. However, these results must be confirmed on a larger prospective cohort with longer follow‐up to see if patients experience late relapse.

Safety data were reassuring; there was no mortality related to the MT. We observed no cases of GVHD during MT treatment. CRS were mainly grade 1–2; we observed only 1 grade 3 and no grade 4. All CRS were easily manageable, and all were resolved in a few days. These results are in accordance with those already reported in previous MT studies.

The occurrence order of mutations must be interpreted with caution because our analysis was based on a small and heterogeneous cohort of AML, MDS and CMML patients, with different disease statuses and with diverse previous treatments. First mutations to appear were involved in the spliceosome complex and DNA methylation, which is coherent with founder mutations reported in the literature [26]. Last significant mutations to appear were *TP53, KRAS*, and *NPM1*, *NPM1* previously described as a late event in AML evolution [26].

Variation of mutations involved in methylation, chromatin modification, and spliceosome complex did not seem to be associated with response nor relapse. We observed the rise of the *TP53* mutated clone in 2 patients with an increase of *TP53* VAF, suggesting that *TP53* may be the vector of progression of the haemopathy. Order of mutations onset confirmed that *TP53* was one of the newest mutations to appear. Clonal evolution was studied in AML patients treated by MT by Sung et al. and they also showed that in patients with refractory AML associated with *TP53*, the variant frequency either remained stable or increased [27]. *TP53* mutated myeloid malignancies are often associated with therapy‐related haemopathy and have poor outcomes, with a higher rate of relapse after standard treatment. Treating *TP53* mutated patients has been a challenge, even with the development of novel therapies. ASCT remains for now one of the only therapies that really improve outcomes, even though OS after ASCT has been estimated to 35% at 1 year [28].

Several strategies are under investigation to improve the outcome of elder or comorbid patients with high‐risk myeloid malignancies: developing alternative consolidation/maintenance treatment, refining criteria of ineligibility to ASCT, continuing to adapt ASCT to frailer patients and finding ways to separate the Graft vs. Leukaemia (GvL) from the GvH effects.

Our results suggest that MT can be a potential consolidation therapy in patients with myeloid malignancies contraindicated to conventional ASCT, especially if they are already in CR. A randomised phase III trial is mandatory to further validate the efficacy and safety of MT for AML patients achieving CR who cannot undergo ASCT. HMA consolidation, especially oral AZA [3, 5], has also proven its efficacy and feasibility in this indication, with a median OS of 24.7 months vs. 14.8 months (*p* < 0.001) compared to simple surveillance. Comparing the two approaches in a randomised trial would be interesting.

Recently the International microtransplant interest group has published an expert consensus on microtransplantation [29]. As patients older than 74 years do not exhibit increased toxicity following microtransplantation, the consensus states that microtransplantation should be considered as a maintenance therapy. Regarding AML subgroups, the consensus recommended considering microtransplantation for standard and adverse AML. The experts reminded that, as demonstrated in our study, even patients with adverse AML exhibited a high CR rate and therefore should not be excluded from benefiting from microtransplantations. The consensus offered some preferred conditioning regimens before microtransplantation depending on the timing of the process (induction or post remission maintenance). In our study, most patients received either azacitidine or venetoclax plus azacitidine as recommended for unfit patients.

Eventually, future studies will need to decipher the complex immune mechanisms underlying the host versus leukaemia and graft versus leukaemia effects.

## 5 Conclusion

Our study shows that MT is a safe consolidation treatment in elderly patients with AML, MDS, and CMML ineligible for conventional ASCT and can provide sustained and deep remission even in high‐risk patients. MT leads to clonal selection with founder mutations not being impacted by MT, regardless of response. Relapse or progression during treatment was mostly driven by a *TP53* subclone.

## Author Contributions

**R. Sammut:** conceptualization (equal), data curation (equal), formal analysis (equal), methodology (equal), writing – original draft (equal), writing – review and editing (equal). **L. Fenwarth:** data curation (equal), writing – review and editing (equal). **A. Pelissier:** formal analysis (equal). **A. Marceau:** data curation (equal). **N. Duployez:** data curation (equal), writing – review and editing (equal). **S. Benachour:** writing – review and editing (equal). **B. Dadone:** writing – review and editing (equal). **T. Cluzeau:** supervision (equal), validation (equal), writing – review and editing (equal). **M. Loschi:** conceptualization (equal), supervision (equal), validation (equal), writing – review and editing (equal).

## Ethics Statement

No ethical committee was required given the retrospective nature of the study and according to the French legislation.

## Consent

All patients gave informed and written consent for data collection and for use in its analyses in accordance with the Declaration of Helsinki.

## Conflicts of Interest

The authors declare no conflicts of interest.

## Reproducing

Any reproducing of the article requires authorisation from the corresponding author.

## Supporting information


**Figure S1.** Mutations VAF variation, S1‐A: mutations VAF variation in patient in CR at last time point, S1‐B: mutations VAF variation in patient with stable disease at last time point, S1‐C: mutations VAF variation in patient in progression at last time point, Abbreviations: VAF variant allele frequency; CR complete response.
**Figure S2:** Fish plot of clonal evolution, Patient 2: AML in constant CR after MT, Patient 15: CMML in progression after MT, Abbreviations: AML acute myeloid leukaemia; CR complete response; MT microtransplantation; CMML.

## Data Availability

The anonymized data collected for this study are available from the corresponding author, upon reasonable request.
