# Supplementary figures and images for: Clonal Evolution of Myeloid Malignancies Treated With Microtransplantation: A Single‐Centre Experience
Source: J Cell Mol Med. 2025 Mar 24;29(6):e70520. doi: 10.1111/jcmm.70520 (PMC11932058; doi:10.1111/jcmm.70520)

**Supplementary data**


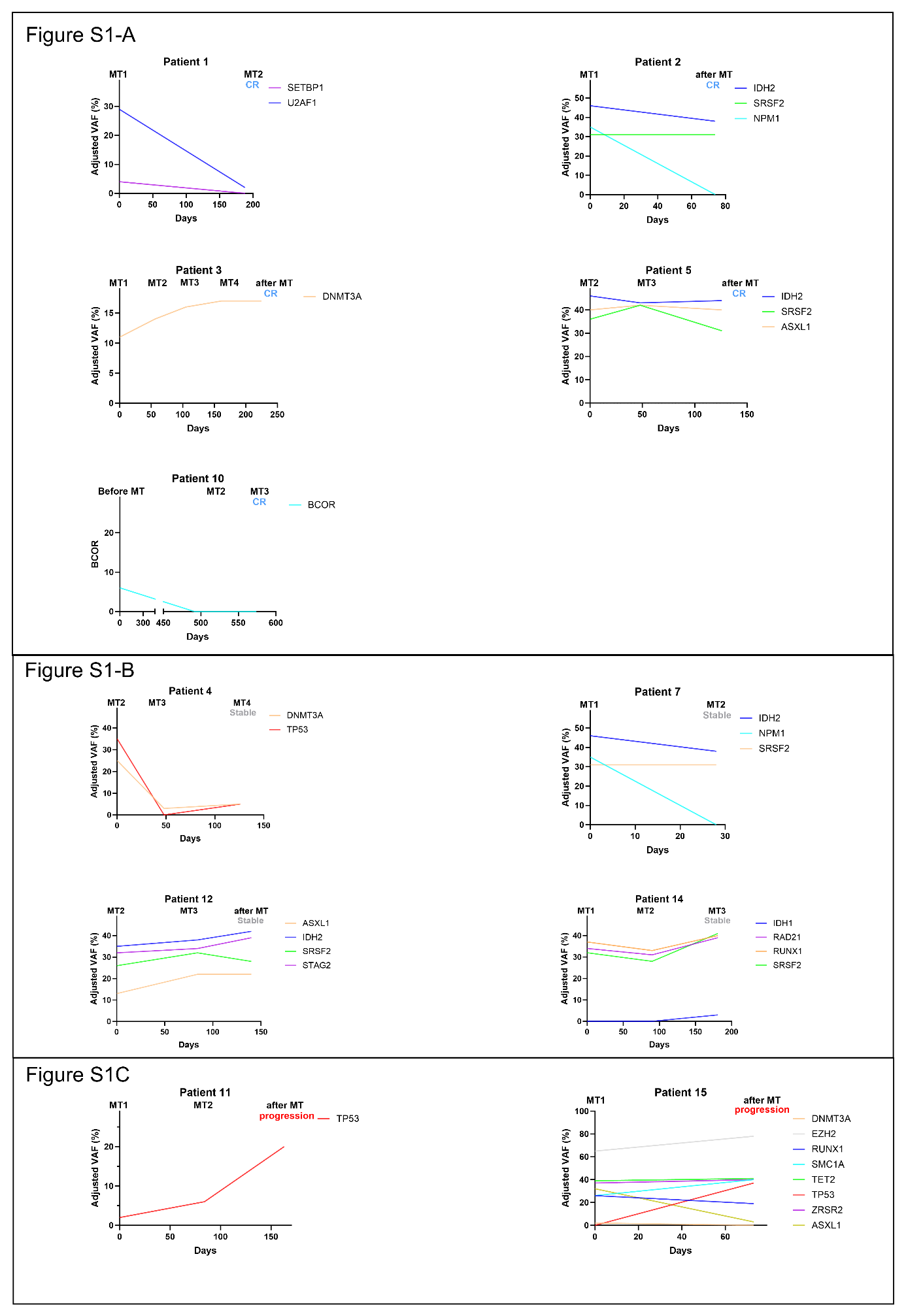


S1-C

S1-B

S1-A


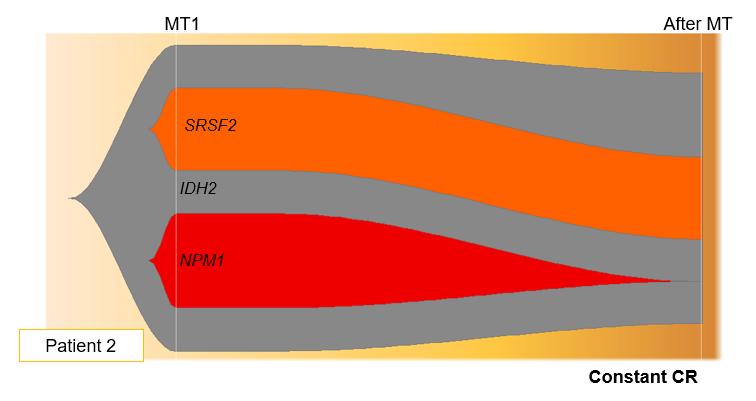
**
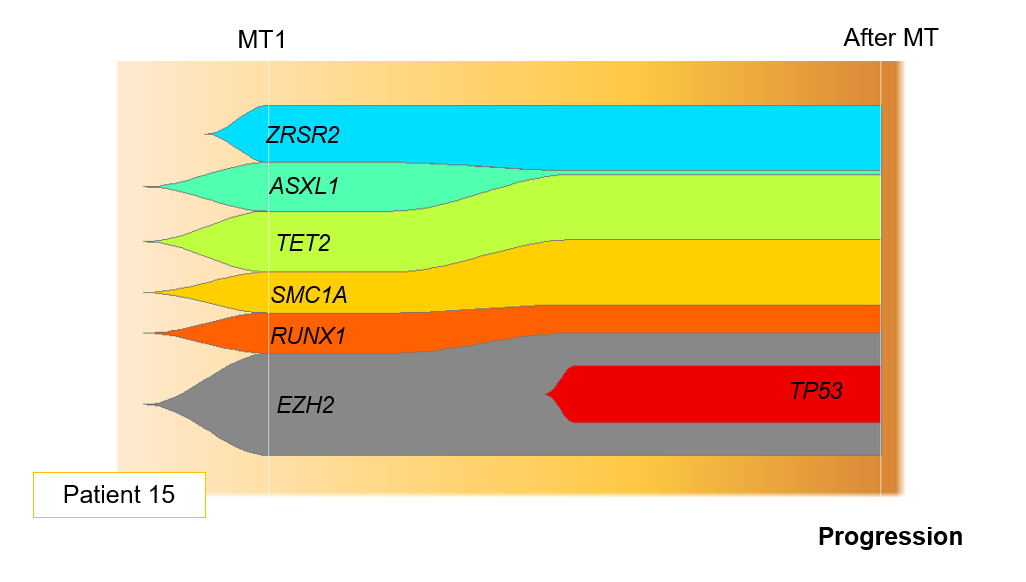
**

S2

Supplement: Supplementary file 1 — Figure S1. Mutations VAF variation, S1‐A: mutations VAF variation in patient in CR at last time point, S1‐B: mutations VAF variation in patient with stable disease at last time point, S1‐C: mutations VAF variation in patient in progression at last time point, Abbreviations: VAF variant allele frequency; CR complete response. Figure S2: Fish plot of clonal evolution, Patient 2: AML in constant CR after MT, Patient 15: CMML in progression after MT, Abbreviations: AML acute myeloid leukaemia; CR complete response; MT microtransplantation; CMML. [file JCMM-29-e70520-s001.docx]
